# 

**Published:** 1984-10

**Authors:** 


Contents

103. Editorial

104. Undergraduate General Practice Teaching in the University of Bristol
Robin Philip

107. Splenic Cyst, the case against Splenectomy
A. A. Shandall, J. M. Frappel and L. R. Celestin

111. Vascular compression of the duodenum, operative and non-operative treatment
A. P. Corfield and R. E. May

114. The Radiology of Tumours of the Pelvis
P. Goddard, S. Scott and E. R. Davies

120. From our Correspondents

124. From our Foreign Correspondent

129. Surgical Club of South West England

133. South West Orthopaedic Club

136. Bristol Medical Care

138. Obituary, Dr Douglas Gawn

139. Book Review—Exotica, W. St C. Symers

140. Programmes of Societies

141. Examination results

